# Elevated Progesterone Levels on the Day of Oocyte Maturation May Affect Top Quality Embryo IVF Cycles

**DOI:** 10.1371/journal.pone.0145895

**Published:** 2016-01-08

**Authors:** Bo Huang, Xinling Ren, Li Wu, Lixia Zhu, Bei Xu, Yufeng Li, Jihui Ai, Lei Jin

**Affiliations:** Reproductive Medicine Center, Tongji Hospital, Tongji Medical College, Huazhong University of Science and Technology, Wuhan, People's Republic of China; Institute of Zoology, Chinese Academy of Sciences, CHINA

## Abstract

In contrast to the impact of elevated progesterone on endometrial receptivity, the data on whether increased progesterone levels affects the quality of embryos is still limited. This study retrospectively enrolled 4,236 fresh *in vitro* fertilization (IVF) cycles and sought to determine whether increased progesterone is associated with adverse outcomes with regard to top quality embryos (TQE). The results showed that the TQE rate significantly correlated with progesterone levels on the day of human chorionic gonadotropin (hCG) trigger (*P* = 0.009). Multivariate linear regression analysis of factors related to the TQE rate, in conventional IVF cycles, showed that the TQE rate was negatively associated with progesterone concentration on the day of hCG (OR was -1.658, 95% CI: -2.806 to -0.510, *P* = 0.005). When the serum progesterone level was within the interval 2.0–2.5 ng/ml, the TQE rate was significantly lower (*P* <0.05) than when the progesterone level was < 1.0 ng/ml; similar results were obtained for serum progesterone levels >2.5 ng/ml. Then, we choose a progesterone level at 1.5ng/ml, 2.0 ng/ml and 2.5 ng/ml as cut-off points to verify this result. We found that the TQE rate was significantly different (*P* <0.05) between serum progesterone levels < 2.0 ng/ml and >2.0 ng/ml. In conclusion, the results of this study clearly demonstrated a negative effect of elevated progesterone levels on the day of hCG trigger, on TQE rate, regardless of the basal FSH, the total gonadotropin, the age of the woman, or the time of ovarian stimulation. These data demonstrate that elevated progesterone levels (>2.0 ng/ml) before oocyte maturation were consistently detrimental to the oocyte.

## Introduction

During conventional *in vitro* fertilization (IVF) cycles, progesterone elevation on the day of human chorionic gonadotropin (hCG) administration refers to rising P levels in the absence of either premature luteinization or a luteinizing hormone (LH) surge [1]. Although the premature luteinization is suppressed by gonadotropin-releasing hormone (GnRH) analogues, early rises in progesterone levels still occur in 5%–50% of all down-regulated IVF cycles [2–4]. The impact of premature progesterone elevation on ART-cycle outcomes has been a subject of some debate in the last two decades [5–7].

In recent years, several large trials and meta-analyses have suggested a negative impact of elevated progesterone on pregnancy rates in GnRH antagonist cycles [8–12]. Most research has reported that elevated progesterone had an adverse impact on the endometrial environment of fresh cycles, leading to a decrease in pregnancy rates. However, to the embryo-endometrial cross-dialog, the embryo quality is as important as endometrial receptivity. Thus, another possibility is that the elevated progesterone has negative effects on the quality of the oocyte or resulting embryo. For this hypothesis, there remains no consensus. First concerns that elevated progesterone is associated with the quality of embryos were raised in 1993 and 1994 [13, 14]. The initial findings were that an elevated serum progesterone level on the day of hCG administration does not adversely affect the quality of oocytes and the resulting embryos [10]. For these studies, the authors used the “usable embryo” as the research subject to determine the impact of elevated progesterone on the quality of embryos. We consider the “usable embryo” as too wide a definition to find a negative effect from elevated progesterone levels. It is well known, that the top quality embryo (TQE) has a direct correlation with the quality of oocyte and IVF cycle outcomes [15, 16]. Thus, we believe that the TQE might be negatively affected by early increase in progesterone.

Nevertheless, with respect to the impact of elevated progesterone on endometrial receptivity, the data in question of whether the presence of increased progesterone levels affects the quality of embryos is still limited. Therefore, the present study sought to determine whether increase in progesterone is associated with an adverse outcome with regard to TQEs.

## Materials and Methods

### Study Design

This was a retrospective, cohort analysis of 4,236 routine fresh IVF cycles in which serum progesterone levels were measured on the day of hCG administration. All patients were treated at the Reproductive Medicine Center of Tongji Hospital between January 2014 and December 2014 and gave written informed consent to participate. The ethics committee of Tongji Hospital approved this study. Patient information was anonymous with no identifiers at the time of data analysis used. All patients in this study underwent routine long GnRH agonist IVF-ET clinical treatment at our center, and no additional intervention was performed.

### Patients

All patients that underwent a fresh IVF cycle during the periods in which serum P levels were measured on the day of hCG administration were included in the analysis. To avoid the impact of male factor infertility on embryo development, exclusion criteria were intracytoplasmic sperm injection (ICSI) cycles and donor oocyte cycles.

### Protocol for Ovarian stimulation

Patients underwent ovarian stimulation according to a long GnRH agonist protocol, as described elsewhere [3]. In general, pituitary suppression was achieved by injection of GnRH agonist (Decapeptyl [Ferrin] or Diphereline [Ipsen] starting in the midluteal phase of the preceding cycle. When pituitary desensitization was confirmed, ovarian stimulation was initiated by intramuscularly administering recombinant FSH (Gonal-F [Serono] or Puregon [MSD]). Recombinant hCG (250 mg; Ovidrel; Serono) was administered to trigger ovulation when two leading follicles reached a mean diameter of 18 mm. Oocytes were retrieved transvaginally 34–36 hours after hCG administration.

### Hormone measurements

The details for hormone measurement have been described previously [3]. Briefly, Serum progesterone and E_2_ levels were measured on the day of hCG administration. The samples were determined using a microparticle enzyme immunoassay (Axsym System, Advia Centaur; Siemens) with a sensitivity of 0.21 ng/mL.

### Embryo Culture and Embryo Grading

The methods used for sperm preparation, for IVF and embryo culture, have been described previously [17]. Briefly, semen was collected in sterile containers by masturbation after 3–5 d of sexual abstinence and then maintained at 37°C for 30 min. After liquefaction, samples were analyzed for sperm concentration, motility and morphology according to the World Health Organization criteria. The oocytes were incubated in G-IVF medium (Vitrolife) and fertilized 3 to 4 hours after retrieval. Normal fertilization was defined as zygotes with two pronuclei (2PN). Then, fertilized oocytes were continuously cultured in G1 medium for 2 more days. Usually fewer than two best-quality embryos were transferred on day 3 after oocyte retrieval. The additional good-quality embryos were cryopreserved for subsequent FET cycles.

All transferable embryos were assessed on day 2 and 3 for blastomere number and regularity as well as for the presence and volume of cytoplasmic fragmentation [18]. Day 3 embryos with eight equal size cells, no cytoplasmic fragments, from Day 2 embryos with four equal size cells, no cytoplasmic fragments were defined as TQE.

### Outcome Measure

The TQE rate per 2PN was the major outcome in our study. Clinical pregnancy was defined as the presence of a gestational sac with fetal heart activity on ultrasound examination 5 weeks after oocyte retrieval.

### Statistics

All data analysis was performed using the Statistical Package for Social Sciences (SPSS) version 13.0 (SPSS Inc., USA). Multivariate linear regression analysis was used to study the factors related to TQE rate in IVF cycles. The odds ratio (OR) and 95% confidence interval (CI) of each of the factors were calculated. Between-group data were analyzed using the nonparametric Mann-Whitney U-test tests. To avoid bias in the results by assuming that any relationship between serum progesterone levels and TQE rates may be linear, patients were divided into five distinct groups according to the serum progesterone levels on the day of hCG administration: ≤1.00, 1.00–1.50, 1.50–2.00, 2.00–2.50 and >2.50 ng/mL. These cut-off levels were chosen to provide equal intervals focused around the threshold values employed in previous studies [19–23]. Statistical significance was established at P<0.05.

## Results

A total of 4,236 IVF cycles were included in this study, of which 2,630 day 3 cleavage-stage ET cycles were performed. The average age of the participants was 31.6 years (range 20–47 years). Clinical indications for infertility included tubal factor, ovarian factor, endometriosis, uterine factor and idiopathic infertility. The clinical features and cycle outcomes of enrolled cycles are shown in Table 1.

**Table 1:** Clinical features and cycle outcomes of ovarian stimulation of IVF cycle patients.

*Parameter*	
***No*. *of fresh cycles***	***4*,*236***
***Age (y)***	***31*.*6±5*.*13***
***Percentage of primary infertility (%)***	***42*.*2***
***Duration of infertility (y)***	***4*.*74±3*.*36***
***Percentage of endometriosis patients (%)***	***3*.*2 (136/4*,*236)***
***Percentage of ovarian factor patients (%)***	***10*.*4 (442/4*,*236)***
***Percentage of tubal factor patients (%)***	***76*.*8 (3*,*254/4*,*236)***
***Basal FSH (IU/L)***	***7*.*42±3*.*10***
***No*. *of oocytes retrieved***	***11*.*5±6*.*97***
***Fertilization rate (%)***	***69*.*8±27*.*49***
***Total gonadotropin (IU)***	***1*,*946±789***
***Duration of stimulation (days)***	***9*.*69±1*.*80***
***Peak estradiol (pg/mL)***	***4*,*119±2*,*834***
***Progesterone on hCG day (ng/mL)***	***1*.*04±0*.*71***
***Ongoing pregnancy rate (%)***	***48*.*5(1*,*276/2*,*630)***

Correlation analysis of factors related to TQE rate (Table 2) demonstrated no significant correlation of TQE rate with: patient age (*P* = 0.32); basal FSH (*P* = 0.06); number of oocytes retrieved (*P* = 0.88); fertilization rate (*P* = 0.12); total Gn (*P* = 0.62); duration of ovarian stimulation (*P* = 0.22); peak estradiol level (*P* = 0.68). By contrast, the TQE rate significantly correlated with duration of patient infertility (*P* = 0.01) and progesterone levels on the day of hCG trigger (*P* = 0.009).

**Table 2:** Correlation analysis of factors related to the TQE rate in conventional IVF cycles.

*Parameter*	*TQE rate*	
	*Pearson C*.*C*.	*P value*
***Age***	***-0*.*015***	***NS***
***Basal FSH***	***-0*.*030***	***NS***
***No*. *of oocytes retrieved***	***0*.*002***	***NS***
***Fertilization rate***	***-0*.*024***	***NS***
***Total gonadotropin***	***0*.*008***	***NS***
***Duration of stimulation***	***0*.*019***	***NS***
***Duration of infertility***	***-0*.*040***	*0*.01*
***Peak estradiol***	***0*.*006***	***NS***
***Progesterone on hCG day***	***-0*.*040***	*0*.009**

TQE, top quality embryo; Pearson C.C., Pearson correlation coefficients NS = not significant.

**, Correlation is significant <0.01 level

*, Correlation is significant <0.05 level.

Table 3 shows the multivariate linear regression analysis of factors related to the TQE rate in conventional IVF cycles; the TQE rate was negatively associated with patient infertility time (OR was -0.38, 95% CI: -0.641 to -0.118, *P* = 0.004) and progesterone concentration on the day of hCG (OR was -1.658, 95% CI: -2.806 to -0.510, *P* = 0.005).

**Table 3:** Multivariate analysis of factors related to the TQE rate in conventional IVF cycles.

*Parameter*	*TQE rate*		
	*P value*	*OR*	*95% CI for Exp (B)*
***Duration of infertility***	***0*.*004***	***-0*.*380***	***-0*.*641– -0*.*118***
***Progesterone on hCG day***	***0*.*005***	***-1*.*658***	***-2*.*806– -0*.*510***

TQE, top quality embryo

**Fig 1:**
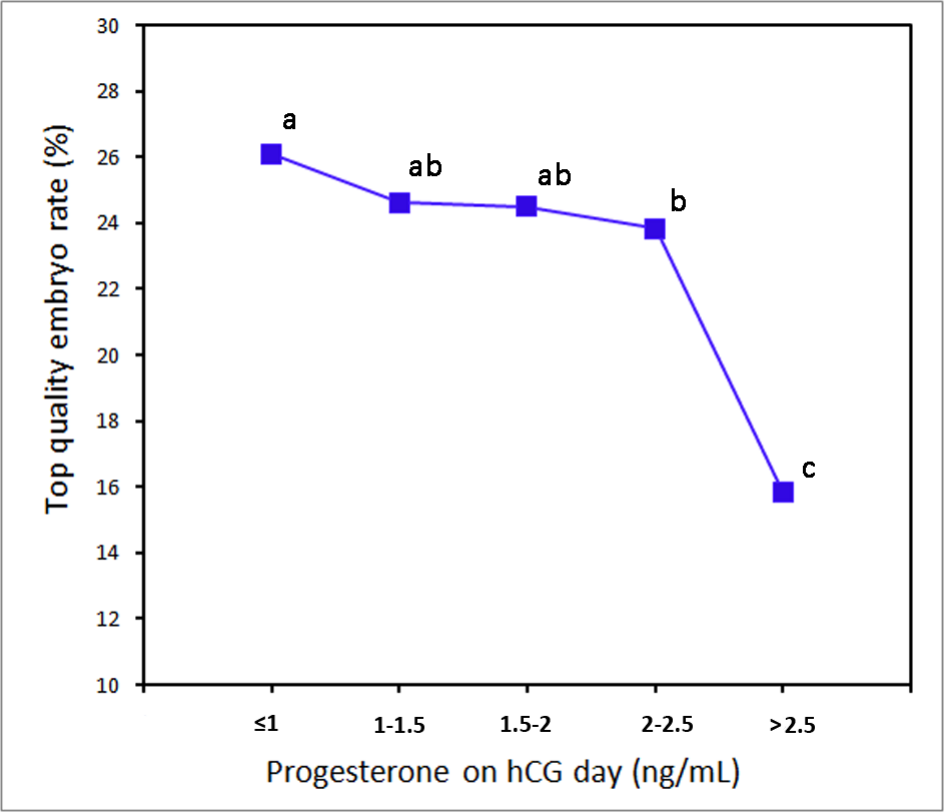
Relationship between serum progesterone concentration and top quality embryo rate. With a decrease in the TQE rate, the serum progesterone concentration increased progressively. When serum progesterone levels were within the interval 2.0–2.5 ng/ml and >2.5 ng/ml, the TQE rate was significantly lower than the progesterone level < 1.0 ng/ml. In each group, values with different superscript letters differ significantly (P<0.05).

**Fig 2:**
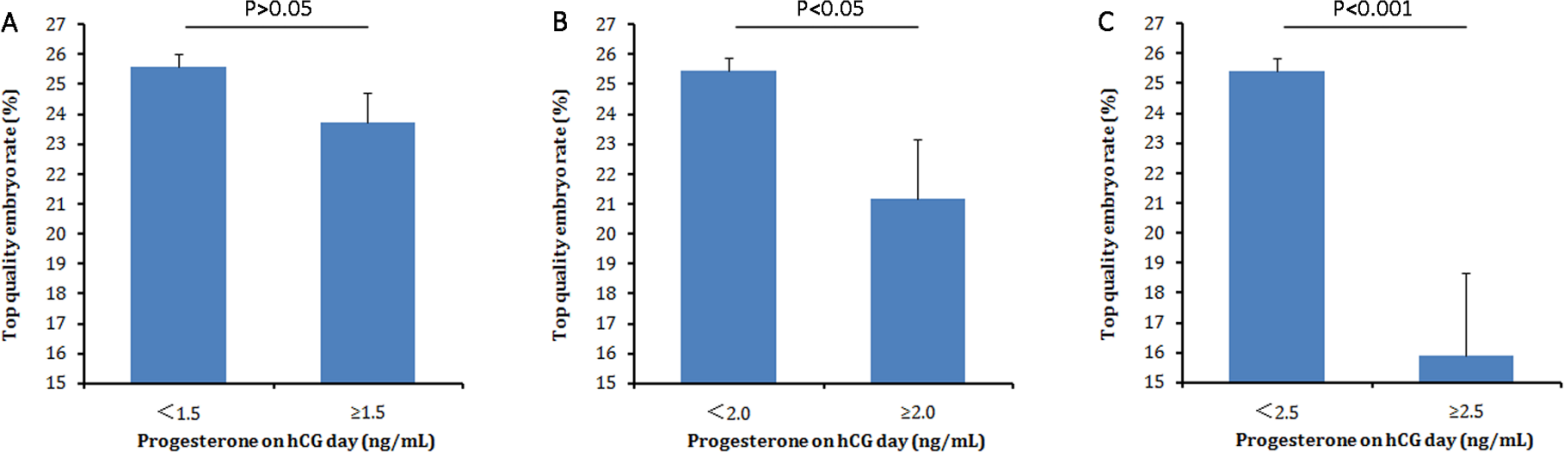
Top quality embryo rate according to different serum progesterone concentration cut-point on the day of hCG administration. (A) progesterone cut-point, 1.5 ng/mL; (B) progesterone cut-point, 2.0 ng/mL; (C) progesterone cut-point, 2.5 ng/mL. The TQE rate was significantly different (*P* <0.05) between serum progesterone levels < 2.0 ng/ml and >2.0 ng/ml.

Fig 1 shows the overall association between serum progesterone levels on the day of hCG administration and the TQE rate. There was a decrease in the TQE rate with progressively higher concentrations of serum progesterone in IVF cycles. In addition, when serum progesterone levels were within the interval 2.0–2.5 ng/ml, the TQE rate was significantly lower (*P* <0.05) than the progesterone level < 1.0 ng/ml; similar results were obtained for serum progesterone levels >2.5 ng/ml. Then, progesterone levels 1.5ng/ml, 2.0 ng/ml and 2.5 ng/ml were chosen as cut-off points to verify the results. Fig 2 shows that the TQE rate was significantly different (*P* <0.05) between serum progesterone levels < 2.0 ng/ml and >2.0 ng/ml.

## Discussion

This retrospective study including 4,236 fresh IVF cycles revealed that elevated serum progesterone on the day of oocyte maturation is associated with a decreased TQE rate. Currently, most investigators have showed that elevated progesterone has a negative impact on the endometrial environment [6, 20, 22, 24]. However, there is limited data available on the impact on the embryo quality, and the TQE rate associated with elevated progesterone.

In the present study, progesterone concentration on the day of hCG was negatively associated with the TQE rate in IVF cycles. The main outcome measure chosen for the current analysis was TQE, not embryo quality or usable embryo quality. We believe that TQE could reflect a small influence from elevated progesterone more accurately. And, we found that when serum progesterone levels were >2.0 ng/ml, the TQE rate was significantly decreased. A recent study found that elevated progesterone on the day of hCG administration was more likely in women with recurrent IVF failure [25]. Women with two or more IVF failures are twice as likely to have elevated progesterone on the day of hCG administration compared to women undergoing their first IVF cycle. Although the authors did not demonstrate why the elevated progesterone on the day of hCG administration was related to recurrent IVF failure, their results indirectly suggested that elevated progesterone on the day of hCG administration may be related to oocyte factors The data of relationship between human oocyte quality and elevated progesterone levels remains limited. However, some investigators reported on the animals’ oocytes. Urrego et al. [26] tested the differences in the expression of OCT-4 and MATER in oocytes and the influence of progesterone concentration on the competence of bovine oocytes. The results indicated that higher levels of oocyte MATER and OCT-4 transcripts and lower progesterone concentration could increase bovine *in vitro* developmental competence. Lower and consistent progesterone concentrations were metabolized during that time, promoting oocyte competence. Oocyte capacitation or cytoplasmic maturation is critical for the oocyte to achieve developmental potential and involves numerous morphologic and biochemical processes. Studies in other animal experiments showed that oocyte competence was regulated by progesterone-responsive genes [27–29].

In conclusion, the results of this study that analyzed 4,236 fresh IVF cycles clearly demonstrated a negative effect of elevated progesterone levels on the day of hCG trigger, on TQE rate, regardless of the basal FSH, the total gonadotropin, the age of the woman, or the time of ovarian stimulation. These data demonstrate that elevated progesterone levels (>2.0 ng/ml) before oocyte maturation were consistently detrimental to the oocyte. However, the mechanism through which elevated progesterone levels causes adverse effects require further study.

## Supporting Information

S1 Text Language edit details. (DOCX)
