# Supplementary material for: Elevated Progesterone Levels on the Day of Oocyte Maturation May Affect Top Quality Embryo IVF Cycles
Source: PLoS One. 2016 Jan 8;11(1):e0145895. doi: 10.1371/journal.pone.0145895 (PMC4706317; doi:10.1371/journal.pone.0145895)
Supplement: S1 Text — (DOCX) [file pone.0145895.s001.docx]

**According to your suggestion, the entire document was edited for proper English language, grammar, punctuation, spelling, and overall style by a qualified English speaking editor at**[**www.mededit.net**](http://www.mededit.net/) **(No. HB110915). Thank you for your kind suggestion.**

**Thanks for your professional and conscientious suggestion, and we hope that we have addressed your concerns.**
